# Manipulating the odds: The effects of Machiavellianism and construal level on cheating behavior

**DOI:** 10.1371/journal.pone.0224526

**Published:** 2019-11-14

**Authors:** Mariela E. Jaffé, Rainer Greifeneder, Marc-André Reinhard

**Affiliations:** 1 Center for Social Psychology, University of Basel, Basel, Switzerland; 2 Chair of Social Psychology, University of Kassel, Kassel, Germany; Western Sydney University, AUSTRALIA

## Abstract

Values, beliefs, and traits differ across individuals, and these concepts might impact whether individuals choose to engage in (dis)honest behavior. This project focuses on interindividual differences in Machiavellianism, which is defined as a tendency toward cynicism and manipulativeness, and the belief that the ends justify the means. We hypothesized that trait Machiavellianism would predict dishonest behavior. Furthermore, we speculated that some situations are more conducive than others for Machiavellianism to translate into behavior. In particular, Construal Level Theory holds that individuals construe social situations on a concrete level, or an abstract level, and that an abstract construal level triggers values and value-related traits to be more influential on behavior. Against this background, we hypothesized that differences in Machiavellianism produce differences in dishonest monetary behavior when situations are construed abstractly. Four studies tested these considerations by asking participants to toss a coin and self-report the toss’ outcome. Inconsistent with our theorizing, we did not find that higher Machiavellianism is consistently associated with a higher self-reported probability of receiving an individual bonus. We also did not find consistent support that higher Machiavellianism is associated with cheating under abstract compared to concrete construal.

## Introduction

Dishonest, deceitful, and fraudulent behaviors may cause severe damage on the individual, group, and societal level [1,2]. In today’s world, there is no shortage of political and economic scandals, and many of these are characterized by persons in power abusing their opportunities to benefit themselves or a select group. A prominent example is the recent “Dieselgate” affair [3], where Volkswagen was accused of having intentionally programmed diesel engines to activate emission controls only during laboratory testing (the diesel engines therefore appeared “cleaner” than they actually were), which caused severe financial harm to customers and stakeholders, and negatively affected the environment in unknown dimensions. Dishonesty was also a major concern in the 2016 US election and in the 2016 BREXIT campaign, during which terms such as “alternative facts” or “post-truth” emerged and thrived. Given that honesty is one of the cardinal values in our society, this picture is rather puzzling. On the one hand, one may speculate that behavior is not guided by the value of honesty. On the other hand, it is possible that honesty is still guiding behavior, but that different, more egoistic values dominate the behavior of powerful politicians and managers. This project touches on both speculations by suggesting that dishonest behavior surfaces when egoistic cognitions are particularly strong and furthermore tests whether values and value-related traits may guide behavior under some circumstances more than others. More specifically, we suggest that a high level of Machiavellianism [4] may result in dishonest behavior. Furthermore, we speculate that this tendency is especially pronounced when the dishonest behavior is self-serving and when individuals have an abstract representation of the situation, which is particularly likely in conditions of power [5,6]. To delineate this hypothesis, we start our review of the literature by focusing on values of virtue and vice. Prior literature has treated Machiavellianism as a belief [7,8], closely related to social values [9], but most frequently as a personality trait [10]. While values and traits are distinct concepts, they share the common signature of intra-individually enduring influence. We emphasize this common signature, but generally opt for the term Machiavellian *personality trait* [11] in what follows, consistent with the majority of prior research. At the same time, when referring to specific prior contributions, we stick to their theoretical framework and terminology. Part of our reasoning is derived from the strong literature on values and here we build on using the respective terms.

### Values of virtue and vice that guide behavior

Values are concepts that pertain to desirable behaviors and transcend specific situations. Values guide the selection or evaluation of behaviors [12], and predict a broad range of meaningful decisions and behaviors, e.g., [13,14]. Schwartz [15], for example, describes that value systems may be used to predict voting behavior in national elections, interpersonal cooperation, and readiness for social contact with outgroup members. One key model regarding values was established by Schwartz [12], who suggested 10 basic human values: universalism, self-direction, stimulation, hedonism, achievement, power, security, conformity, tradition, and benevolence. Per the definition, all values pertain to desirable end states [12], both on the individual and on the societal level. Schwartz’s values could therefore be considered as values of virtue. To illustrate one value in exemplary fashion, consider the value of security, which is driven by the motivational goal of safety, harmony, and stability of society, of relationships, and of the self [12]. Security values result in the pursuit of individual interests (e.g., by staying healthy), but also collective interests (e.g., protecting national security).

Next to these values of virtue, there are also more “negative” and “dark” values and personality traits, which reflect more egoistic motives that individuals might pursue. These traits pertain to less desirable end states, if not for the individual then on the societal level. Individuals, for example, differ with respect to Machiavellianism [4]. Machiavellianism is described as a “tendency toward Machiavellian behavior, in particular cynicism, a penchant for manipulativeness, and the belief that the end sanctifies the means” [16] (p. 54). Machiavellianism is generally measured as the extent to which respondents agree with statements taken from Machiavelli’s original books [4]. Some contributions refer to Machiavellianism as a belief, e.g., [7,8], which is closely related to social values such as moral flexibility and the devaluation of collective interests [9]. Other contributions consider Machiavellianism as one of the three personality traits of the Dark Triad, next to narcissism and psychopathy [10]. All three dark traits entail a socially malevolent character with behavior tendencies toward self-promotion, emotional coldness, duplicity, and aggressiveness [10]. Machiavellianism, for example, has been linked to active deception [17] and unethical behavior [18]. All dark traits are primarily linked to the pursuit of individual and not collective interests. Interestingly, recent research has also documented that Machiavellians also engage in prosocial behavior that benefits their organizations. The underlying reason for this, however, is to increase their chances of benefitting economically and professionally from their employer, which again is a selfish motive [19], see also [20].

### The power of the situation: When do values guide behavior?

Values are intra-individually enduring concepts. Their impact, however, might depend on the specific social situation, as prior research has illustrated. For example, values have been shown to exert a stronger impact when individuals actually focus on their standards of behavior. Consider evidence reported by Beaman, Klentz, Diener, and Svanum [21], who placed a mirror behind a bowl of Halloween candy and asked children to take one piece only. The presence of a mirror led to decreased transgression rates in those children who mentioned their names and addresses before, in that they were less likely to take more than one piece of candy. The authors argue that the mirror increased individuals’ self-awareness, which renders a person more likely to focus on her/his standards of behavior. Similarly, Diener and Wallbom [22] showed that college students tended to cheat less on an intelligence test when they observed their reflection in a mirror. Providing further corroborating evidence, Mazar, Amir, and Ariely [23] showed that making moral standards more accessible through situational cues (e.g., by asking participants to either write down the Ten Commandments or books they had read in high school) made individuals behave more honestly. Kleinlogel, Dietz, and Antonakis [24] applied these findings to personality traits and showed that the factor Honesty-Humility, the tendency to be sincere, fair, and modest [25], predicted cheating behavior. Individuals low on Honesty-Humility cheated more when exposed to immoral (compared to moral) primes, while individuals high on Honesty-Humility were less affected by situational primes.

Findings such as these attest that situational cues influence whether values impact behavior. Situations can motivate individuals with a given tendency to act out on this tendency (“press”) [26]. To the extent that values are related to virtues, increasing their impact on behavior appears highly laudable. However, if values are related to vices, this strategy could also backfire. In particular, if the situational cues increase the salience of “dark” values, individuals might be more likely to manipulate and cheat.

### How does construal level influence the impact of values on behavior?

Eyal, Sagristano, Trope, Liberman, and Chaiken [27] showed that values exerted a greater impact on how individuals plan their distant future than their near future. The researchers assessed participants’ values and asked them to read eleven vignettes of hypothetical situations that presented a behavior that was consistent with one of the values. When planning the distant future, values and behavioral intentions correlated more strongly, while this relation was attenuated when participants planned for the close future [27]. These findings were corroborated by Rixom and Mishra [28], who assumed that individuals generally value honesty, and therefore showed that participants who adopted an abstract construal level compared to a concrete construal level tended to behave more honestly. Reversely, in situations in which dishonesty served the greater good of another party, benevolence in the form of money given to charity was the value in focus, and participants who adopted an abstract construal level therefore tended to be behave less honestly. Presumably, here their behavior was driven by the value of benevolence, and more so under abstract than under concrete construal level. In both cases, participants’ behavior was impacted more strongly by their values when they were primed to adopt an abstract compared to concrete construal level.

These findings can be explained against the background of Construal Level Theory [29–31], which holds that individuals may construe social situations on a high-and-abstract level, or a more low-and-concrete level. Abstract representations contain the gist of situations, meaning superordinate and central information, as well as information on the desirability of situations. By definition, values are abstract and decontextualized, and are therefore hypothesized to serve as high-level behavioral guides [29]. In contrast, concrete representations contain more details and subordinate information, as well as more information regarding the feasibility of actions in situations. To illustrate this differentiation between abstract and concrete, consider a game of basketball [30]: An abstract representation of a game of basketball could be “having fun,” while a more concrete representation could include that the players are children of a certain age. Here, “the higher-level construal disregards the unique features of the event and entails an implicit decision about which features are central to the event and which are peripheral” [30].

The level of construal depends on psychological distance. Psychologically distant objects or events (in regards to time, space, social connection, or likelihood) are construed more abstractly, and psychologically close objects or events more concretely. A basketball game taking place a year from now (versus today) would be more likely to be construed abstractly (concretely). Reversely, because values are abstract and decontextualized, they are expected to be more readily applied to and guide intentions for psychologically distant situations [29], but see [32] for a replication that was only partially successful. Interestingly, because traits have been defined as “probabilistic descriptions of relatively stable patterns of emotion, motivation, cognition, and behavior” [33], they are also rather decontextualized concepts, so that traits may be similarly expected to more likely guide behavior when construal is abstract.

### Dishonesty as a result of an abstract construal level and Machiavellianism

The following general considerations were derived against the background of Construal Level Theory: Individuals’ values and traits impact their (dis)honest behavior, and more so in conditions of abstract construal level. Importantly, if values and traits are related to virtues (e.g., fairness), individuals with high scores should be more likely to show honest behavior, particularly when construing abstractly. If values and traits relate to vices, however, individuals with high scores should be more likely to show dishonest behavior, again particularly when construing abstractly.

From these general considerations we derived the specific hypothesis that individuals with high compared to low scores on Machiavellianism are more likely to show dishonest behavior. We furthermore qualified this hypothesis by speculating that the relationship between Machiavellianism and dishonest behavior is particularly pronounced when construing abstractly. While this contribution focuses on Machiavellianism, one could argue that similar reasoning may relate to other values, beliefs, and personality traits, too, that are related to dishonest behavior such as narcissism, e.g., [10], belief in a just world [34], or belief in a competitive-jungle world [35]. We will later return to these considerations.

We tested our Machiavellianism hypotheses in four studies. The first one took an exploratory approach. Here we assessed a variety of constructs to measure values and traits. We then tested our hypothesis and analyzed whether values and traits explain dishonest behavior in general (Hypothesis 1) and more so in conditions of abstract compared to concrete construal (Hypothesis 2). The second study was of a confirmatory nature. Here, we focused on Machiavellianism and replicated the finding that individual differences in Machiavellianism explain honest versus dishonest behavior under abstract but not under concrete construal level. Study 3 and 4 were conducted to replicate the results from Study 2. Finally, data from Studies 2, 3, and 4 were pooled to conduct a raw data meta-analysis. An IRB Approval by the Institutional Review Board of the Faculty of Psychology from the University of Basel has been obtained for this research project. Participants’ consent has been obtained in a written manner throughout all studies.

## Study 1

Study 1 explored the general notion that negatively connoted personality traits (e.g., Machiavellianism, narcissism, and others) impact behavior (i.e., cheating), and more so in conditions of abstract construal. To this end, we assessed individuals’ Machiavellianism scores as well as several other values, beliefs, and traits, which have proven to predict a multitude of different behaviors.

We included the 10 basic human values [12], given their predominance in the research field. Moreover, we included individuals’ belief in a just world. This belief is associated with reciprocal behavior, and we assumed that it could be related to honest behavior, because strong believers in a just world might fear the negative fate that would befall someone who cheats [36]. Furthermore, we assessed individuals belief in a just world [34], which refers to individuals’ belief that their environment is a just and orderly place where people usually get what they deserve [37]. Edlund, Sagarin, and Johnson [36], for example, have shown that belief in a just world is associated with reciprocal behavior. Individuals who were stronger in the belief in a just world engaged in more reciprocal behavior. Presumably, individuals then might also be less likely to engage in dishonest behavior.

We also included a set of personality traits that are more “dark” and potentially linked to more dishonest behavior. Machiavellianism and narcissism are both associated with tendencies towards self-promotion [10]. Whereas Machiavellianism is described as the belief that the end sanctifies the means [16], narcissism is associated with facets such as entitlement or dominance [10]. The association between both personality traits and dishonest behavior have been investigated, and findings indicate that Machiavellianism could be linked to the propensity to lie in different contexts [17,38,39]. We did not assess the complete Dark Triad. One reason not to include psychopathy is that psychopathy predicts a wider range of socially undesirable behavior beyond dishonesty.

We also assessed individuals’ belief in a competitive-jungle world, which is defined as the belief that “the social world is a competitive jungle characterized by a ruthless, amoral struggle for resources and power in which might is right and winning is everything” [35]. Some of the items clearly indicate that dishonesty might be an appropriate behavior (such as “You know that most people are out to “screw” you, so you have to get them first when you get the chance”), although this scale is more related to the study of prejudice, ethnocentrism, and intergroup hostility [35].

All variables included so far are likely predictive of (dis-)honest behavior. However, we also wished to test for discriminant validity and therefore included a measure that should not predict dishonest behavior. In doing so, we wished to demonstrate that our outcome measure is sensitive to variables related to cheating and would not display associations with variables that are unrelated to cheating behavior. Against this background, we elected to assess the personality trait extraversion as the last variable, as it is, to our knowledge, not associated with dishonest behavior, e.g., [40], but see [41] for findings that deviate from the results of the meta analysis.

All in all, Study 1 explored the research question of whether negatively connoted traits impact dishonest behavior, and whether they do so more in conditions of abstract compared to concrete construal. We relied upon a setting in which dishonest behavior increases the likelihood of gaining a bonus payment, either for the individual (self-serving) or for a charitable organization (other-serving). The self-serving versus other-serving variation was conceptually inspired by the work of Rixom and Mishra [28], who implemented a condition in which cheating would serve a “good” charitable organization compared to a “bad” charitable organization. These authors found that cheating increased under an abstract construal level when a “good” organization would profit, but decreased when a “bad” organization would profit. Against this background, it appeared fruitful to vary the beneficiaries of cheating, and we decided to do so by introducing a self-serving and an other-serving condition. We assumed that dark traits translate into dishonest behavior (and more so under abstract construal level), particularly if the dishonest behavior is self-serving. However, in a condition where other people would benefit, this association between Machiavellianism and dishonest behavior should be attenuated or eventually even reverse.

### Method

#### Participants and design

Research within the area of Construal Level Theory indicates the occurrence of medium sizes [42]. With no knowledge of effect sizes focusing on the interplay between construal level and deceptive behavior, we decided to assess 40 participants for each between subjects cell (construal level and bonus recipient) as a rough approximation to achieve sufficient power, resulting in a minimum of 160 participants.

One hundred and sixty four US-American participants (*M*_*age*_ = 32.27 years, *SD* = 11.24) were recruited for a “Personality study” via prolific academic [43] and completed the study by reporting an outcome regarding the coin toss, which served as our main dependent variable, and were therefore included in the analyses. Because men compared to women have been reported to show higher scores on Dark Triad variables [44,45], we recruited male participants only to avoid floor effects among the predictor variables. Participants received £1.25 (approximately US$1.5) as compensation and could gain another £0.5 as a bonus for themselves or charity.

The study used a mixed design with two independent variables that were orthogonally manipulated between participants: Construal Level (abstract vs. concrete) and the recipient of a small monetary bonus (participants vs. a charitable organization), which could be won at the end of the experiment. Participants were randomly assigned to conditions. Furthermore, we assessed individuals’ traits (see Materials). As the dependent variable we assessed the likelihood of participants winning a bonus payment of £0.5 (see Materials and Procedure for the exact setup).

#### Materials and procedure

Participants first gave informed consent, before working on the different parts of the study listed below.

**Assessment of individual differences of participants.** Participants first completed several personality and value scales, such as the Twenty Item Values Inventory [46] with statements such as “S/he believes s/he should always show respect to his/her parents and to older people. It is important to him/her to be obedient” rated on a scale from 1 = *very much like me* to 6 = *not like me at all*. Cronbach’s alpha for these scales was: Conformity = .61, Tradition = .53, Benevolence = .77, Universalism = .77, Self Direction = .59, Stimulation = .70, Hedonism = .75, Achievement = .76, Power = .85, Security = .24. Next, they completed the Competitive Jungle Social World View Scale [35], rating statements such as “Winning is not the first thing; it’s the only thing” on a scale from 1 = *very strongly disagree* to 9 = *very strongly agree* (Cronbach’s alpha = .93). They then completed the Narcissistic Personality Inventory [47], choosing between two options each, for example “I have a natural talent for influencing people” versus “I am not good at influencing people” (Cronbach’s alpha = .90). Participants then worked on the MACH-IV scale to assess Machiavellianism [4], rating statements such as “Never tell anyone the real reason you did something unless it is useful to do so” on a scale from 1 = *strongly disagree* to 5 = *strongly agree* (Cronbach’s alpha = .84). Next, they completed the Multidimensional Belief in a Just World scale [34], rating statements such as “I think that I deserve the reputation I have among the people who know me” on a scale from 1 = *strongly disagree* to 7 = *strongly agree* (Cronbach’s Alpha = .70). Lastly, participants completed one scale to asses Extraversion [48], rating statements such as “I see myself as someone who is talkative” on a scale from 1 = *disagree strongly* to 5 = *agree strongly* (Cronbach’s alpha = .90).

**Manipulation of construal level.** Next, participants completed a construal level manipulation. Participants were randomly assigned to a concrete or abstract construal level condition and asked to either think about why (abstract level) or how (concrete level) they would try to reach an objective of improving and maintaining one’s physical health [49].

**Assessment of cheating behavior.** Subsequently, participants were introduced to a coin toss, with which we assessed cheating behavior on the group level (see S2 Fig for the instructions’ specific wording). As cheating is not a socially desirable behavior, we decided to use a more anonymous and subtle paradigm to detect this behavior. This paradigm was developed from the Randomized Response Technique [50] and has been extensively used in the past to study dishonesty, e.g., [51–53]. In this paradigm, participants are asked to toss a coin (or a die, see e.g. [54] once to determine whether or not they would win a bonus payment and in which they self-report the outcome. They do so at home, and we did not provide any link to an online coin flip animation, as we did not want to raise any suspicion that we might record the outcome. In our instructions participants were informed at the very beginning that the study would require a coin and that they should ensure that they had one beside them. At the end of the study they were then asked to flip the coin and self-report the outcome (see S2 Fig). Winning the bonus was coded as 1, and not winning the bonus was coded as 0. By this means, cheating cannot be detected on the individual level, as it is impossible for the researchers to know whether an individual actually tossed heads or tails. However, on the group level, a significant deviance from chance (.5) can be interpreted as indication of cheating and dishonesty in this group.

Within this setup we varied whether the bonus could be won for either the participants themselves or for a charitable organization, to contrast a self-serving condition (i.e., participants themselves benefit from deceptive behavior) to an other-serving condition (i.e., the charity organization UNICEF could benefit from deceptive behavior). Moreover, for methodological reasons, we counterbalanced whether the bonus was won for tossing heads or tossing tails. After self-reporting the result of the coin toss, participants were thanked and received the code for their compensation.

### Results

**Manipulation check construal level.** To investigate the success of our construal level manipulation, we followed a procedure introduced by Fujita, Trope, Liberman, and Levin-Sagi [55] and asked two independent coders, who were blind to the construal level conditions, to rate the answers of participants’ reasoning on how/why to increase and maintain one’s physical health. If a response served as a means to the end of increasing health (indicating a low construal level), answers were coded as -1. If the response served as an end of the means of increasing health, answers were coded as +1. Answers without a clear relation were coded as 0. Ratings of each participant’s four responses were then summed and averaged across the two raters (*ICC* = .90). This resulted in an index of level of construal with a potential range of– 4 to + 4; higher scores indicate higher levels of construal. Our results attest to the manipulation’s success by indicating that participants primed to an abstract construal level compared to a concrete construal level displayed a more abstract mindset, *M* = 3.14, *SD* = 1.05; *M* = -3.47, *SD* = 0.59; respectively; *t*(125.12) = 49.31, *p* < .001.

**Results on dishonesty.** In total, 118 out of 164 participants reported winning the bonus payment (72%). A binomial test indicated that this was higher than expected by chance (.50), *p* < .001. Theoretically, with a fair coin, half of the participants should have won the bonus, while the other half should not have won the bonus, so that a significant deviance from chance level is an indication of cheating on the group level. We analyzed the cheating behavior following the guidelines of Moshagen and Hilbig [56] using the R package RRreg (for correlation and regression analyses of randomized response data, see [57]). Prevalence of cheating can be estimated by taking into account (a) the baseline probability of winning (.50) and (b) the proportion of participants claiming to win a bonus (for further details, see [56]).

Calculations revealed that within our sample, 44% of participants were estimated to have been dishonest. Within the abstract construal level condition this number increased to 53%, whereas in the concrete condition it was 35%. In the condition where participants received a bonus, this number increased to 47%, and decreased to 41% when the bonus recipient was a charitable organization. Nevertheless, the logistic regression analysis indicated that neither the effect of condition, nor recipient, nor the interaction was significant (all *Likelihood Ratio Tests* < 0.36, *p*s > .552).

Next, we included the different trait measures (mean-centered) in the analysis and calculated separate logistic regressions with construal level (dummy coded with 0 = abstract construal level and 1 = concrete construal level), bonus recipient (dummy coded with 0 = participants as bonus receiver and 1 = charity as bonus receiver) and the respective trait measures on the likelihood of winning the bonus.

**Fig 1:**
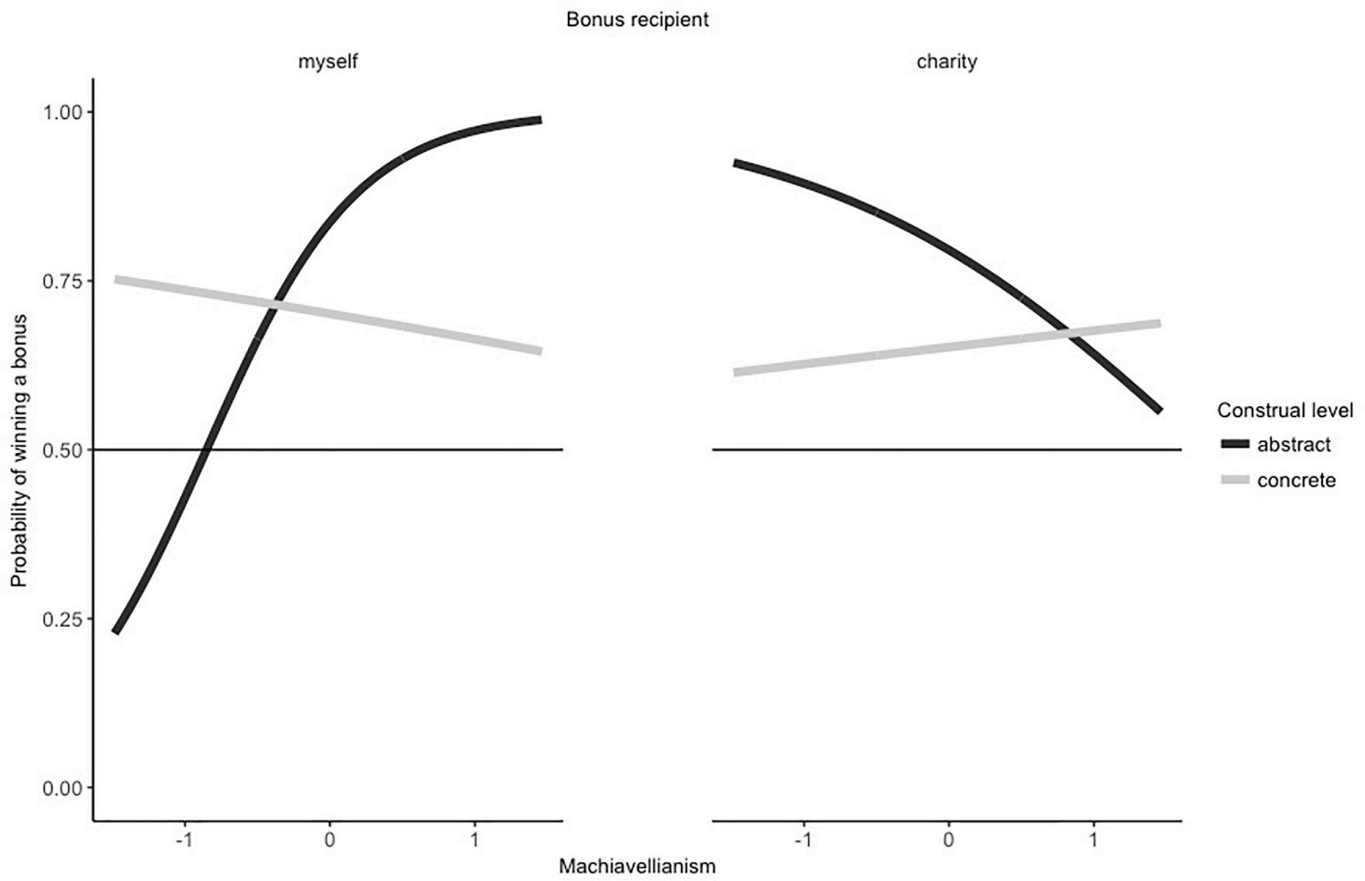
Depiction of the three-way interaction between bonus recipient, construal level, and Machiavellianism scores (mean centered). Figures were created using a logistic regression model.

We first analyzed the impact of the Dark Triad variables Machiavellianism and narcissism. In this model, Machiavellianism significantly predicted cheating behavior, *b* = 6.37, *SE b* = 4.53, *Likelihood Ratio Test* = 8.79, *p* = .003. This effect was qualified by an interaction between bonus recipient and Machiavellianism, *b* = -7.45, *SE b* = 4.69, *Likelihood Ratio Test* = 7.69, *p* = .006 and, in tendency, by the predicted three-way interaction between bonus recipient, construal level, and Machiavellianism, *b* = 8.01, *SE b* = 5.23, *Likelihood Ratio Test* = 3.81, *p* = .051. To disentangle the reported three-way interaction, we calculated correlations between Machiavellianism and the probability of winning the bonus for the four conditions of abstract construal level * participant as bonus recipient (*r* = .38, *t*(40) = 2.60, *p* = .013), concrete construal level * participant as bonus recipient (*r* = -.03, *t*(35) = -0.17, *p* = .864), abstract construal level * charity as bonus recipient (*r* = -.15, *t*(37) = -0.92, *p* = .363), concrete construal level * charity as bonus recipient (*r* = .02, *t*(44) = 0.16, *p* = .877). For participants as bonus recipients, higher scores on Machiavellianism are associated with an increased probability of “winning” the bonus–but only under abstract and not under concrete construal level. For charity as bonus recipients, the interaction pattern appears to be the opposite: When looking at the correlations descriptively, under abstract construal level higher scores on Machiavellianism are associated with a decreased probability of “winning” the bonus, while under concrete construal level the association appears to be weaker. However, although the three-way interaction indicates that the two-way interaction terms differ between the two between-subjects conditions, the two two-way interactions were not significant, *p*s > .066. Fig 1 summarizes the findings.

Narcissism (the total score resulting from the Narcissistic Personality Inventory), in contrast, was not a significant predictor for dishonest behavior. No main or interaction effects reached significance, all *Likelihood Ratio Tests* < 0.86, all *p*s > .354 (see Table 1 for the exact inferential statistics for this and all following regression analyses). Going beyond the Dark Triad, we analyzed the impact of a jungle world view and, turning more towards the values of virtue, belief in a just world. Both concepts did not significantly predict dishonest behavior (results depicted in Table 1). The same applies for the 10 basic human values of Schwartz (results depicted in S1 Fig).

**Table 1:** Summary of Study 1 regression analyses predicting cheating behavior.

		Regression analysis for randomized response data (RReg)			
Value / Trait	Predictor	*b*	*SE b*	*Likelihood Ratio Test*	*p*
MACH-IV Scale	Construal Level	-1.38	1.07	1.69	.193
	Bonus Recipient	-0.64	1.04	0.39	.533
	Machiavellianism	6.37	4.53	8.79	.003
	Construal Level x Bonus Recipient	0.21	1.39	0.02	.878
	Construal Level x Machiavellianism	-6.73	4.90	3.35	.067
	Bonus Recipient x Machiavellianism	-7.45	4.69	7.69	.006
	Construal Level x Bonus Recipient x Machiavellianism	8.01	5.23	3.81	.051
Narcissistic Personality Inventory	Construal Level	-0.44	0.98	0.22	.636
	Bonus Recipient	0.17	0.80	0.05	.832
	Narcissism	2.10	2.80	0.58	.445
	Construal Level x Bonus Recipient	-0.59	1.35	-0.06	1.00
	Construal Level x Narcissism	-3.36	6.18	0.44	.509
	Bonus Recipient x Narcissism	-2.67	3.85	0.49	.483
	Construal Level x Bonus Recipient x Narcissism	6.15	7.54	0.85	.355
Competitive Jungle Social World View (CJSWV)	Construal Level	-0.58	0.87	0.48	.488
	Bonus Recipient	0.12	0.77	0.02	.881
	CJSWV	0.23	0.34	0.48	.489
	Construal Level x Bonus Recipient	-0.47	1.22	0.15	.703
	Construal Level x CJSWV	-0.51	0.76	0.47	.494
	Bonus Recipient x CJSWV	-0.57	0.55	1.14	.287
	Construal Level x Bonus Recipient x CJSWV	0.68	0.95	0.52	.471
Multidimensional Belief in a Just World (MBIAJW)	Construal Level	-0.61	0.71	0.72	.397
	Bonus Recipient	0.07	0.77	0.01	.925
	MBIAJW	-6.37	4.42	5.78	.016
	Construal Level x Bonus Recipient	-	-	-	-
	Construal Level x MBIAJW	6.61	5.25	1.45	.228
	Bonus Recipient x MBIAJW	6.64	4.56	4.18	.041
	Construal Level x Bonus Recipient x MBIAJW	-5.44	5.56	0.83	.364
Value / Trait	Predictor	*b*	*SE b*	*Likelihood Ratio Test*	*p*
Extraversion	Construal Level	-2.17	2.12	3.45	.063
	Bonus Recipient	-0.32	0.77	0.17	.678
	Extraversion	-0.23	0.55	0.18	.672
	Construal Level x Bonus Recipient	-	-	-	-
	Construal Level x Extraversion	-1.47	1.89	0.87	.351
	Bonus Recipient x Extraversion	0.15	0.81	0.03	.858
	Construal Level x Bonus Recipient x Extraversion	4.68	4.54	2.94	.086

As predicted, extraversion was not significantly related to dishonest behavior, all *Likelihood Ratio Tests* < 3.45, all *p*s > .062. Table 1 and S1 Fig summarize the results of all conducted analyses. Importantly, regression analyses for randomized response data revealed numeric instabilities when estimating the full model for Belief in A Just World and Extraversion. We therefore omitted one interaction term (construal level and bonus recipient) that is neither conceptually important nor proved to be empirically relevant in the present study.

### Discussion

This first exploratory study suggests that higher scores of Machiavellianism lead to an increasing likelihood of winning a bonus and therefore supports Hypothesis 1. In line with our theorizing regarding Hypothesis 2, this relation might differ depending on participants’ construal level and the bonus recipient. Most importantly, when participants are able to win a bonus for themselves and construe the task abstractly, a significant positive correlation between Machiavellianism scores and winning probability occurs, attesting to increased cheating behavior. But this correlation drops to zero under concrete construal. The relations between Machiavellianism, construal level, bonus recipient, and dishonest behavior are especially interesting as they seem to be unique for this variable. Having said this, the exploratory nature of Study 1 needs to be acknowledged and addressed in Study 2.

## Study 2

Study 1 provides first support that Machiavellianism predicts dishonest behavior and that when individuals construe abstractly, stronger Machiavellian tendencies increase the probability of winning a bonus in self-serving conditions. However, this first study was exploratory by nature. Study 2 was therefore conducted to replicate these findings in a confirmatory study, focusing on Machiavellianism and the self-serving bonus condition only.

### Method

#### Participants and design

Sample size was determined based on the effect size (differences between slopes for linear bivariate regressions) of Study 1, a power of .90, and an alpha-level of .05, resulting in 242 participants [58]. Two hundred forty-two male US-American participants were recruited for a “Personality study” via prolific academic. Although the requirements of the study indicated that we were only recruiting male participants, two participants indicated their gender as female and one refrained from answering. Because it is unclear whether these data points represent true answers or reflect “misclicking,” we decided to retain these “female” data points in the sample. Excluding them did not change the pattern or significance levels in our Machiavellianism-regression analysis.

Individuals who had participated in Study 1 were not able to participate. Two hundred forty-two participants (*M*_*age*_ = 30.21 years, *SD* = 9.84) completed the study by self-reporting the coin toss’ outcome, which served as our main dependent variable. One person indicated that his data should not be used, and we therefore excluded this participant from further analyses. Participants received £0.66 (approximately US$0.66) as compensation and could gain another £0.5 as a bonus for themselves.

The study used a mixed design with Construal Level (abstract vs. concrete, dummy coded with 0 = abstract construal and 1 = concrete construal) as an independent between factor with random assignment. Individuals’ trait level of Machiavellianism served as a second independent variable. The likelihood of participants winning a bonus of £0.5 for themselves was the dependent variable.

#### Materials and procedure

Participants first gave informed consent, before working on the different parts of the study. The setup was similar to Study 1 with the following changes. First, all participants could win the bonus payment for themselves. Second, participants completed the MACH-IV scales only, as Study 2 was designed to confirm the stronger association between Machiavellianism under abstract compared to concrete construal level found in Study 1. Cronbach’s alpha for the MACH-IV scale was .75.

### Results

**Manipulation check construal level.** To investigate the success of our construal level manipulation, we followed the procedure described in Study 1. The ICC across our two judges was .92. Our results indicate that participants primed to an abstract construal level compared to a concrete construal level displayed a more abstract mindset, *M* = 3.35, *SD* = 0.98; *M* = -3.35, *SD* = 0.86; respectively; *t*(239) = 56.28, *p* < .001, thus attesting to the manipulation’s success.

**Fig 2:**
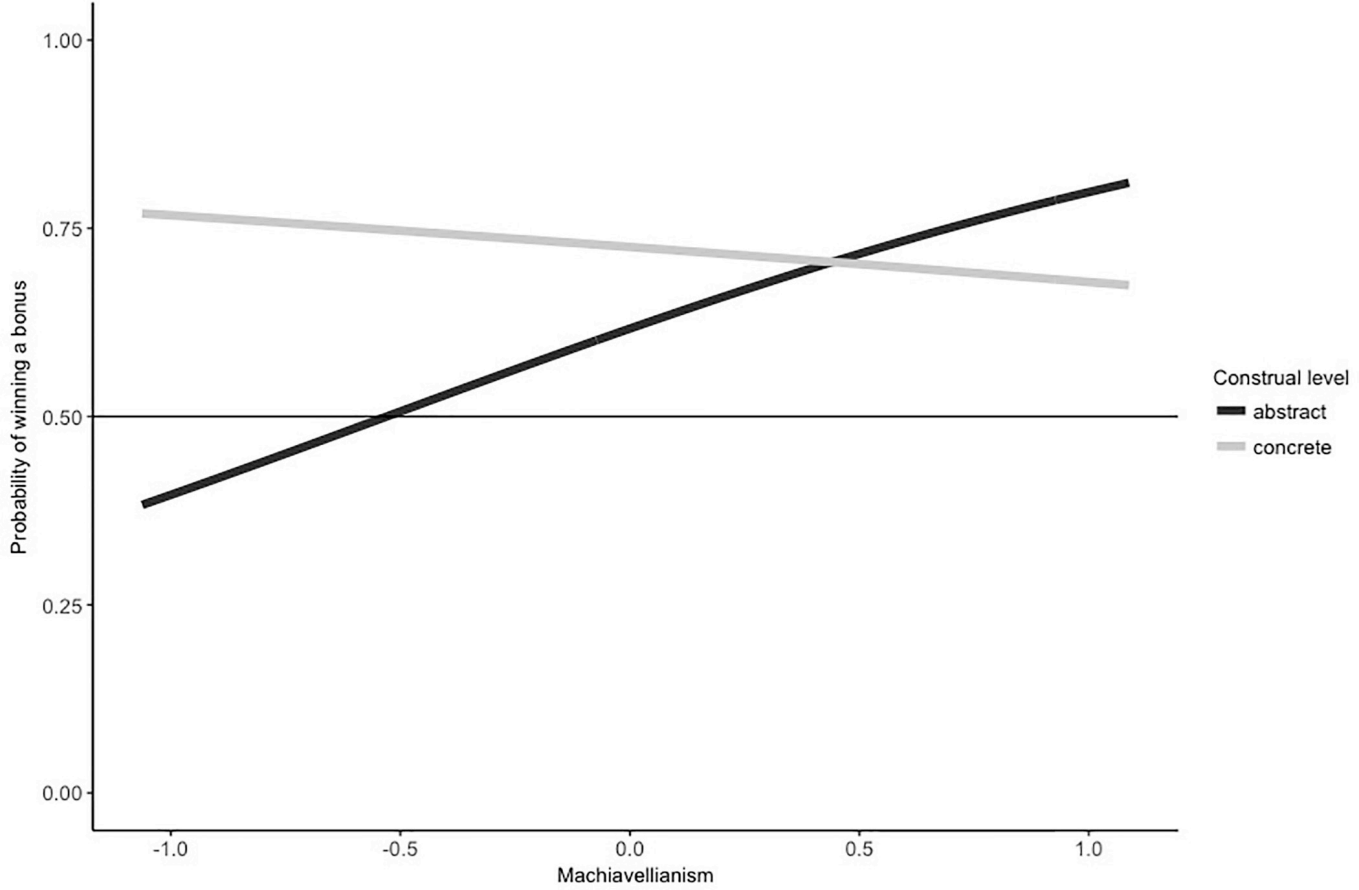
Depiction of the two-way interaction between construal level and Machiavellianism (mean centered). Figures were created by using a logistic regression model.

**Results on dishonesty.** In total, 159 out of 241 participants reported winning the bonus payment (66%). A binomial test indicates that this proportion of wins was higher than expected by chance (.50), *p* < .001. We analyzed the cheating behavior following the guidelines of Moshagen and Hilbig [56] using the R package RRreg [57]. Calculations revealed that within our sample, 32% of participants were estimated to have been dishonest. Within the abstract construal condition this number decreased to only 19%, while in the concrete construal condition it was 44%. The logistic regression analysis indicated that Machiavellianism (mean centered) significantly predicts dishonesty in this model (*b* = 4.03, *SE b* = 2.56, *Likelihood Ratio Test* = 5.14, *p* = .023). Furthermore, construal level was a significant predictor, (*b* = 1.63, *SE b* = 1.19, *Likelihood Ratio Test* = 4.58, *p* = .032), indicating that concrete construal participants were more likely to act dishonestly than participants in the abstract construal condition. Interestingly, the predicted two-way interaction between the two predictors is significant, *b* = -4.34, *SE b* = 2.66, *Likelihood Ratio Test* = 5.04, *p* = .025 (see Fig 2). Study 2 therefore replicates the pattern observed in Study 1.

To disentangle the two-way interaction, we calculated correlations between Machiavellianism and the probability of winning the bonus for the two construal level conditions. In the abstract condition, higher Machiavellianism scores were associated with a higher probability of winning, *r* = .17, *t*(117) = 1.88, *p* = .062, while Machiavellianism was not associated with a higher probability of winning in the concrete construal condition, *r* = -.04, *t*(120) = -0.46, *p* = .650. The significant two-way interaction indicates that these two slopes are different from each other.

### Discussion

The results from Study 2 replicate the findings from Study 1 in a confirmatory, well-powered setting with a new sample. Stronger Machiavellianism scores were associated with a higher likelihood for dishonest behavior. Furthermore, this association was more pronounced under abstract construal level, while there was no such relation under concrete construal. As predicted by Construal Level Theory, the personality trait Machiavellianism impacted behavior more strongly when construing on a more abstract level.

Interestingly, in Study 2, cheating was more pronounced in conditions of concrete construal, while no statistically significant main effect was observed in Study 1. Our theorizing did not suggest ex ante predictions for main effects. This may possibly be a result of statistical power, that is, the ex ante likelihood of detecting effects if they are there, which was much higher in Study 2 than Study 1. While we had not specified ex ante predications for main effects, it is interesting to note that a main effect of construal level is in line with findings reported by other authors, e.g., [28]. To gauge this main effect’s reliability, we conducted Study 3.

## Study 3

Study 3 was an exact replication of Study 2 with the objective to learn more about the effects’ generalizability. The design, materials, as well as the procedure were identical to Study 2. Therefore, respective descriptions only mention differences or highlights.

### Method

#### Participants

Sample size was determined based on the effect size (slope between Machiavellianism and probability of winning a bonus in the abstract condition) of Study 2, a power of .80, and an alpha-level of .05, resulting in 212 participants for the abstract condition (and 424 for the entire sample) [58]. We added 10% as a buffer to this number for potential exclusions (see below) and collected data from 486 participants. Male US-American participants were recruited for a “Personality study” via prolific academic. Four hundred eighty-six participants (*M*_*age*_ = 33.69 years, *SD* = 11.35) completed the study by self-reporting the coin toss’ outcome, which served as our main dependent variable. Eight persons indicated that their data should not be used, and we therefore excluded these participants from further analyses. Furthermore, five participants stated their gender as female (despite only male individuals being admitted to the study), and were therefore excluded from further analyses, resulting in a sample of 473 participants. Participants received approximately US$ 1.01 (£0.77) as compensation and could gain another US$ 0.50 (£0.38) as a bonus for themselves.

#### Design, materials, and procedure

The design of Study 3, the materials and procedure were identical to Study 2. Cronbach’s alpha for the MACH-IV scale was .80 in Study 3.

### Results

**Manipulation check construal level.** To investigate the success of our construal level manipulation, we followed the procedure described in Study 1. While looking at the coding of the answers, we observed that four participants had not completed the manipulation properly and two more had obviously participated twice. We excluded these six participants from further analyses. The ICC across two judges was .80. Results indicate that participants primed to an abstract construal level compared to a concrete construal level displayed a more abstract mindset, *M* = 2.68, *SD* = 1.86; *M* = -3.82, *SD* = 0.49; respectively; *t*(262.15) = 51.41, *p* < .001, thus attesting to the manipulation’s success.

**Results on dishonesty.** In total, 317 out of 467 participants reported winning the bonus payment (68%). A binomial test indicates that this proportion of wins was higher than expected by chance (.50), *p* < .001. Using the R package RRreg [57], 36% of participants were estimated to have been dishonest. Within the abstract construal condition this number increased to 40%, while in the concrete construal condition it was 32%. The logistic regression analysis indicated no significant main or interaction effects. Neither Machiavellianism (mean centered; *b* = 0.26, *SE b* = 0.52, *Likelihood Ratio Test* = 0.24, *p* = .623), nor construal level (*b* = -0.34, *SE b* = 0.38, *Likelihood Ratio Test* = 0.79, *p* = .373), or the interaction between the two predictor variables (*b* = -0.81, *SE b* = 0.80, *Likelihood Ratio Test* = 1.04, *p* = .309) significantly predicted the likelihood of winning a bonus.

### Discussion

Study 3 does not replicate the pattern observed in Study 2. Neither the main effect for Machiavellianism, nor the main effect for construal level, nor the interaction between Machiavellianism and construal level were significant. Study 3 therefore does not confirm our hypotheses.

To understand what may account for these differences, we investigated potential differences between the samples in Studies 2 and 3. One aspect that caught our attention was participants’ mean age, which in Study 2 was lower than in Study 3 (M_Study2_ = 30.21 versus M_Study3_ = 33.69). Although this difference might seem negligible, it may prove important, because participant age and our key variable of interest, Machiavellianism, are negatively correlated, *r*(239) = -0.25, *p* < .001 in Study 2 and *r*(465) = -0.21, *p* < .001 in Study 3. This negative association has also been found in other work, e.g., [59]; Christie [60] further suggests that Machiavellianism scores might increase in adolescence (age 10 to age 16 or 17) and gradually decrease after the age of 40. We exploratorily analyzed only data from participants older than 22 and younger than 45 years old (+/- 1 SD from the mean) and included age (centered) in the regression model. With this restricted (and likely underpowered) dataset, we find effects consistent with the hypotheses, namely for Machiavellianism (mean centered; *b* = 1.10, *SE b* = 0.89, *Likelihood Ratio Test* = 1.59, *p* = .207), construal level (*b* = -0.09, *SE b* = 0.51, *Likelihood Ratio Test* = 0.03, *p* = .860), and the interaction between the two predictor variables (*b* = -2.29, *SE b* = 1.24, *Likelihood Ratio Test* = 3.68, *p* = .055). These results indicate that a certain level of Machiavellianism scores may prove critical to uncover the predicted associations between Machiavellianism and cheating. However, because this analysis was conducted ex post, it needs to be treated with caution and a further replication appeared important.

## Study 4

Study 3 did not replicate the results from Study 2. However, based on the exploratory results, one might speculate that a certain level of Machiavellianism scores is required to investigate the potential effects of Machiavellianism and construal level on dishonest behavior. Informed by the exploratory analysis of Study 3, we decided to conduct a further well-powered replication study that is identical to Studies 2 and 3, but limits participant age to the range of 18 to 40. We preregistered the entire study on aspredicted.org: http://aspredicted.org/blind.php?x=ub9y9w.

### Method

#### Participants

Sample size was determined based on the effect size of Study 2, a power of .80, and an alpha-level of .05, resulting in a total of 424 participants [58]. To ensure that the sample size would meet the sample size in Study 3, we increased that number to 484 and added 10% as buffer to this number for potential exclusions (see below). In total we collected data from 532 participants. Male US-American participants were recruited for a “Personality study” via prolific academic and could only participate if they were between 18 and 40 years old and had not participated in the previous study. Although we had preregistered the age restriction before collecting the data, by a technical mistake, the filter was not applied at the beginning of the collection phase. Therefore, we had to collect data from another 128 participants. Data was not analyzed before completion of data collection; and all analyses reported here include only data from the preregistered sample of participants aged 18 to 40 years.

Five hundred thirty-three participants (*M*_*age*_ = 27.97 years, *SD* = 5.92) completed the study by self-reporting the coin toss’ outcome, which served as our main dependent variable. Nine persons indicated that their data should not be used, and therefore were excluded from further analyses. Furthermore, five participants stated their gender as female (despite only male individuals being admitted to the study) and three participants did not complete the manipulation successfully (see below), and were therefore excluded from analyses, resulting in a final sample of 516 participants. Participants received approximately US$ 0.90 (£0.69) as compensation and could gain another US$ 0.50 (£0.38) as a bonus for themselves.

#### Design, materials, and procedure

The design of Study 4, the materials and procedure were identical to Study 2. Cronbach’s alpha for the MACH-IV scale was .76.

### Results

**Manipulation check construal level.** To investigate the success of our construal level manipulation, we followed the procedure described in Study 1. While looking at the coding of the answers, we found 3 participants had not completed the manipulation properly and were therefore excluded from further analyses. The ICC across two judges was .98. Results indicate that participants primed to an abstract construal level compared to a concrete construal level displayed a more abstract mindset, *M* = 3.82, *SD* = 0.87; *M* = -3.88, *SD* = 0.55; respectively; *t*(514) = 121.02, *p* < .001, thus attesting to the manipulation’s success.

**Results on dishonesty.** In total, 357 out of 516 participants reported winning the bonus payment (69%). A binomial test indicates that this proportion of wins was higher than expected by chance (.50), *p* < .001. Using the R package RRreg [57], 38% of participants were estimated to have been dishonest. Within the abstract construal condition this number dropped to 35%, while in the concrete construal condition it was 41%. The logistic regression analysis indicated no significant main or interaction effects. Neither Machiavellianism (mean centered; *b* = 0.71, *SE b* = 0.77, *Likelihood Ratio Test* = 0.90, *p* = .343), nor construal level (*b* = 0.23, *SE b* = 0.36, *Likelihood Ratio Test* = 0.41, *p* = .522), or the interaction between the two predictor variables (*b* = 0.03, *SE b* = 0.95, *Likelihood Ratio Test* = 0.00, *p* = .997) significantly predicted the likelihood of winning a bonus. Including age in the analysis did not change the resulting patterns.

### Discussion

The results from Study 4 do not support our hypotheses that Machiavellianism (Hypothesis 1) and the interaction between Machiavellianism and construal level (Hypothesis 2) predict dishonest behavior. As in Study 3, the results in Study 4 do not replicate the findings from Study 2, although we used an identical design and collected data from a more age-homogeneous sample. Drawing conclusions appears difficult, as Studies 1 and 2 offer support, while Studies 3 and 4 do not. To gain insight into overall effects, we decided to pool the data from Studies 2, 3, and 4 into one overall data file and run a raw data analysis.

## Integrative (meta) analysis across studies 2, 3, and 4

In order to test our hypotheses across the three identical studies in this manuscript, we pooled the data of Studies 2, 3, and 4 (total *n* = 1224) into one dataset and report the results below. In total, 833 out of 1224 participants reported winning the bonus payment (68%). A binomial test indicates that this proportion of wins was higher than expected by chance (.50), *p* < .001. Using the R package RRreg [57], 36% of participants were estimated to have been dishonest. Within the abstract construal condition this number dropped to 34%, while in the concrete construal condition it was 38% (see Table 2 for a summary of these estimates across all studies).

**Table 2:** Summary of estimates of proportions of dishonest participants across Studies and construal level conditions.

	Estimate of proportion of dishonest participants				
	Study 1	Study 2	Study 3	Study 4	Integrative Analysis
Overall	44%	32%	36%	38%	36%
Abstract construal	53%	19%	40%	35%	34%
Concrete construal	35%	44%	32%	41%	38%

The logistic regression analysis indicated no significant main or interaction effects. Neither Machiavellianism (mean centered; *b* = 0.73, *SE b* = 0.45, *Likelihood Ratio Test* = 2.80, *p* = .094), nor construal level (*b* = 0.20, *SE b* = 0.24, *Likelihood Ratio Test* = 0.70, *p* = .401), nor the interaction between the two predictor variables (*b* = -0.66, *SE b* = 0.57, *Likelihood Ratio Test* = 1.40, *p* = .236) significantly predicted the likelihood of winning a bonus. We summarize effect sizes, which have been calculated in line with the procedure reported in [61] in Table 3, which also provides an overview of the effect sizes for Studies 2, 3, and 4.

**Table 3:** Overview of effect size estimates (odds ratio) for model 1 (M1, original model) and model 2 (M2, including age).

	Odds Ratio (OR)							
Predictors	Study 2		Study 3		Study 4		Integrative Analysis	
	M1	M2	M1	M2	M1	M2	M1	M2
Construal Level (Dummy coded)	5.11	5.57	0.71	0.74	1.26	1.29	1.22	1.26
Machiavellianism (z-standardized)	5.37	5.75	1.13	1.08	1.36	1.33	1.39	1.36
Age (z-standardized)	-	1.11	-	0.78	-	0.85	-	0.84
Construal Level x Machiavellianism	0.16	0.16	0.68	0.67	1.00	1.01	0.74	0.73

*Note*. Odds ratios of OR = 1.25, 1.67, and 2.50 (and the inverse values 0.80, 0.60, and 0.40) for z-standardized predictors as small, medium, and large effects, respectively, see [61].

As we found a significant negative correlation between age and Machiavellianism in previous studies, we exploratorily investigated this association. Across the pooled data that includes the age-restricted sample of Study 4, Machiavellianism and age correlated negatively, *r*(1222) = -0.19, *p* < .001. We therefore again explored whether including age (mean centered) as main effect in the regression analysis would change the resulting pattern. Interestingly, it does. Neither Machiavellianism (mean centered; *b* = 0.68, *SE b* = 0.47, *Likelihood Ratio Test* = 2.12, *p* = .140), nor construal level (*b* = 0.23, *SE b* = 0.24, *Likelihood Ratio Test* = 0.92, *p* = .338), nor age (*b* = -0.02, *SE b* = 0.01, *Likelihood Ratio Test* = 2.06, *p* = .151) significantly predict the likelihood of winning a bonus. Furthermore, the interaction between Machiavellianism and construal level is not significant, (*b* = -0.71, *SE b* = 0.59, *Likelihood Ratio Test* = 1.50, *p* = .221). To exploratorily disentangle the interaction term, we looked at the correlations between Machiavellianism and winning probability separately for the high versus low construal conditions. Results indicate that in the high level condition, Machiavellianism and likelihood of winning correlate to *r*(593) = .06, *p* = .140 and in the low level condition to *r*(627) = .01, *p* = .843.

It should be noted that we did not pre-register the analysis with age as a covariate. These results, therefore, need to be treated with caution. Nevertheless, they allow for the speculation that the hypothesized relationship between Machiavellianism and cheating as a function of construal level additionally depends on participant age, most likely because Machiavellianism scores and participant age are negatively correlated.

## General discussion

In this manuscript we investigated the impact of Machiavellianism on (dis)honest behavior using a coin toss paradigm. Results across four studies suggest that Machiavellianism did not significantly predict dishonest behavior. We also tested whether the relation between Machiavellianism and dishonest behavior was more pronounced in situations where individuals adopted a more abstract compared to concrete construal level, and when they personally benefited from the dishonest behavior. Results across studies were inconsistent and thus do not provide support for this interaction hypothesis. We summarize and discuss these findings in what follows.

Study 1 provided initial support for the hypothesized interaction between Machiavellianism and construal level on dishonest behavior, but was associated with two methodological caveats. First, because Study 1 initially tested the present hypothesis, we had no good estimate for the expected effect size and therefore relied on a rule of thumb as a sampling criterion. Ex post it appears that Study 1 was statistically underpowered, which may have resulted in the situation that associations were descriptively in line with our hypothesis, but did not reach significance. Furthermore, Study 1 was exploratory in nature, which together with the presumably low power, resulted in the requirement of a second, confirmatory Study 2. This Study 2 provided full support for the predicted main effect of Machiavellianism and dishonest behavior as well as for the interaction between Machiavellianism and construal level, showing that the association between Machiavellianism and dishonest behavior was stronger under abstract compared to concrete construal level. However, in Study 2 an unpredicted main effect for construal level on cheating was observed, and it appeared desirable to run well-powered follow-up studies to gauge reliability. Unfortunately, Studies 3 and 4 offer inconsistent evidence. Both studies did not provide support for the previously observed association between Machiavellianism and dishonest behavior. Furthermore, they did not support the interaction hypothesis between Machiavellianism and construal level on dishonest behavior. This picture is then strengthened by pooling the data from Studies 2, 3, and 4 and conducting a raw data meta analysis. Here again, we did not find support for Hypothesis 1 or Hypothesis 2.

In Study 3, exploratory analyses showed that participant age played an unexpectedly important role, as it was negatively correlated with Machiavellianism, see also [59,60]. Future research may fruitfully rely on these exploratory findings as a starting point.

### Future research

Besides the interesting impact of age, future research could also check whether the negative association between Machiavellianism and participant age is associated to the MACH-IV scale used in this study, or generalizes to other scales, such as the Dirty Dozen [62], the Short Dark Triad (SD3) [63], or the Machiavellianism Personality Scale (MPS) [64]. It could also be commendable to develop and include measures reflecting the construct’s multifaceted conceptualization [65,66], to better understand differences and the differential impact of the facets of Machiavellianism. Here, we relied on the MACH-IV scale, which is assumed to be the “most important scale” [45], and persisted with this choice in Studies 3 and 4 to provide exact replications. If the negative association between Machiavellianism and participant age was specific to the MACH-IV scale, this choice could prove ill-informed in hindsight.

One limitation of our studies is that we did not include other potentially interesting variables, such as Honesty-Humility [25] or psychopathy [10]. Future research could therefore focus on these and other values and traits that have been linked to dishonest behavior and assess how they interact with individuals’ construal level. Moreover, future studies could introduce a temporal gap between the assessment of traits and the assessment of dishonest behavior. Such a gap would ensure against the potential caveat that assessing traits renders these unusually salient. If true, unusually high salience could artificially strengthen or reduce the traits’ impact on behavior.

In this contribution, we hypothesized that Machiavellianism is linked to behavior more strongly under abstract construal level, but did not find support for this hypothesis. This null-finding may dovetail with other evidence suggesting that the original finding of values on judgment as a function of psychological distance (related to abstract construal [67]) was replicated using some, but not all psychological distance manipulations [32]. Alper [68] describes differential effects as well, showing that in some cases abstract construal increased deception while in other cases it decreased deception. These findings suggest that our null-finding may be specific to the construal level manipulation we used, and may not necessarily generalize to other construal level manipulations. We opted for a manipulation that allowed manipulating construal level through goal priming, without affecting the related concept of psychological distance [69]. Furthermore, this manipulation has been well studied and used across a multitude of construal level studies, just to name a few, [28,70–76]. However, researchers have also noted that the manipulation has certain disadvantages, as it elicits different thought content in the different conditions [73]. Therefore, in retrospect, running exact replications in Studies 3 and 4 may not have been the best decision, as using different paradigms could have allowed for more far-reaching conclusions. Future studies could use alternatives such as the category versus exemplar task [55], or turn to manipulations that manipulate construal level by changing psychological distance, for example, by asking to think about the future versus today, a stranger versus yourself, a far-away place versus right here, or a very unlikely versus a very likely scenario, for an overview, see [29]. At the same time and concerning this manuscript, however, we believe that running exact replications allows for a rather firm conclusion that using the paradigms as we did does not uncover support for the suggested hypotheses.

It may also be interesting to replicate the current studies with different levels of bonus payments and compensations for the participants. In our studies, the bonus was quite high, with compensation resulting in a payment increase of, for example, 42% in Study 1 and 75% in Study 2. Arguably, such high bonuses might have invited higher levels of dishonesty in our overall sample. At the same time, it is interesting to note that reported winning probabilities in our study were only slightly lower than those in studies using similar designs, e.g., [53].

Last but not least, it may be fruitful to sample from populations that do not only include male participants, to investigate the effect across participant populations. Our choice was informed by prior research [44,45]; nevertheless, conclusions beyond the tested population can be more rigorous if other populations are tested, too.

## Conclusion

In this manuscript we investigated the impact of Machiavellianism on (dis)honest behavior using a coin toss paradigm. We did not find support for the assumption that Machiavellianism significantly predicts dishonest behavior. Furthermore, we did not find support for an interaction between Machiavellianism and construal level on dishonest behavior. However, our research indicates that age might play a role when investigating the interaction between Machiavellianism and construal level and future research could fruitfully build on this exploratory finding to better understand predictors of dishonest behavior.

## Supporting information

S1 Fig Summary of Study 1 regression analyses on the 10 basic human values (Schwartz, 1992) predicting cheating behavior. (DOCX)

S2 Fig Screenshot of the instructions for the coin flip task. (DOCX)
