# Supplementary material for: Manipulating the odds: The effects of Machiavellianism and construal level on cheating behavior
Source: PLoS One. 2019 Nov 14;14(11):e0224526. doi: 10.1371/journal.pone.0224526 (PMC6855464; doi:10.1371/journal.pone.0224526)
Supplement: S1 Fig — (DOCX) [file pone.0224526.s001.docx]

**Summary of Study 1 regression analyses on the 10 basic human values (Schwartz, 1992) predicting cheating behavior.**

|  |  | Regression analysis for randomized response data (RReg) | | | |
| --- | --- | --- | --- | --- | --- |
| Value | Predictor | *b* | *SE b* | *Likelihood Ratio Test* | *p* |
| Conformity  (10 basic values) | Construal Level | -2.15 | 1.88 | 2.32 | .128 |
|  | Bonus Recipient | 0.27 | 0.80 | 0.12 | .733 |
|  | Conformity | 0.43 | 0.50 | 0.78 | .378 |
|  | Construal Level x Bonus Recipient | 0.04 | 2.51 | 0.00 | .988 |
|  | Construal Level x Conformity | 2.49 | 2.24 | 1.90 | .168 |
|  | Bonus Recipient x Conformity | -1.20 | 0.72 | 3.11 | .078 |
|  | Construal Level x Bonus Recipient x Conformity | -2.98 | 2.63 | 1.48 | .224 |
| Tradition  (10 basic values) | Construal Level | -1.73 | 2.02 | 2.76 | .097 |
|  | Bonus Recipient | 0.05 | 0.78 | 0.00 | .946 |
|  | Tradition | 0.49 | 0.45 | 1.38 | .240 |
|  | Construal Level x Bonus Recipient | - | - | - | - |
|  | Construal Level x Tradition | 0.97 | 1.97 | 0.29 | .590 |
|  | Bonus Recipient x Tradition | -0.73 | 0.70 | 1.11 | .291 |
|  | Construal Level x Bonus Recipient x Tradition | 0.38 | 1.34 | 0.08 | .776 |
| Benevolence  (10 basic values) | Construal Level | -0.50 | 0.87 | 0.33 | .564 |
|  | Bonus Recipient | 0.03 | 0.79 | 0.00 | .974 |
|  | Benevolence | 0.72 | 0.62 | 1.64 | .201 |
|  | Construal Level x Bonus Recipient | -0.53 | 1.26 | 0.18 | .673 |
|  | Construal Level x Benevolence | -0.08 | 0.86 | 0.01 | .922 |
|  | Bonus Recipient x Benevolence | -0.84 | 0.85 | 1.05 | .306 |
|  | Construal Level x Bonus Recipient x Benevolence | 0.54 | 1.27 | 0.18 | .674 |
| Universalism  (10 basic values) | Construal Level | 2.35 | 2.66 | 1.02 | .312 |
|  | Bonus Recipient | 2.70 | 2.83 | 1.12 | .289 |
|  | Universalism | 1.21 | 092 | 3.14 | .076 |
|  | Construal Level x Bonus Recipient | -4.27 | 3.56 | 1.69 | .194 |
|  | Construal Level x Universalism | -1.28 | 1.07 | 2.08 | .149 |
|  | Bonus Recipient x Universalism | -1.20 | 1.10 | 1.58 | .209 |
|  | Construal Level x Bonus Recipient x Universalism | 1.68 | 1.35 | 1.83 | .176 |
|  |  | Regression analysis for randomized response data (RReg) | | | |
| Value | Predictor | *b* | *SE b* | *Likelihood Ratio Test* | *p* |
| Self Direction  (10 basic values) | Construal Level | -1.52 | 1.13 | 3.71 | .054 |
|  | Bonus Recipient | -0.57 | 0.80 | 0.52 | .470 |
|  | Self Direction | 1.42 | 0.91 | 3.20 | .074 |
|  | Construal Level x Bonus Recipient | - | - | - | - |
|  | Construal Level x Self Direction | -0.62 | 1.35 | 0.16 | .686 |
|  | Bonus Recipient x Self Direction | -1.79 | 1.11 | 3.21 | .073 |
|  | Construal Level x Bonus Recipient x Self Direction | -1.44 | 2.55 | 0.42 | .517 |
| Stimulation  (10 basic values) | Construal Level | -0.73 | 1.04 | 0.61 | .436 |
|  | Bonus Recipient | 0.17 | 0.79 | 0.05 | .826 |
|  | Stimulation | 0.08 | 0.57 | 0.02 | .889 |
|  | Construal Level x Bonus Recipient | -0.50 | 1.41 | 0.12 | .732 |
|  | Construal Level x Stimulation | 0.40 | 0.97 | 0.19 | .663 |
|  | Bonus Recipient x Stimulation | -0.64 | 0.77 | 0.72 | .396 |
|  | Construal Level x Bonus Recipient x Stimulation | -0.70 | 1.27 | 0.32 | .569 |
| Hedonism  (10 basic values) | Construal Level | -0.48 | 0.82 | 0.36 | .550 |
|  | Bonus Recipient | 0.07 | 0.76 | 0.01 | .925 |
|  | Hedonism | 0.03 | 0.43 | 0.00 | .950 |
|  | Construal Level x Bonus Recipient | -1.07 | 1.51 | 0.58 | .447 |
|  | Construal Level x Hedonism | 0.01 | 0.73 | 0.00 | .991 |
|  | Bonus Recipient x Hedonism | -0.11 | 0.67 | 0.03 | .874 |
|  | Construal Level x Bonus Recipient x Hedonism | -1.40 | 1.39 | 1.17 | .279 |
| Achievement  (10 basic values) | Construal Level | -1.26 | 1.01 | 2.27 | .132 |
|  | Bonus Recipient | 0.08 | 0.79 | 0.01 | .920 |
|  | Achievement | 0.83 | 0.65 | 2.25 | .133 |
|  | Construal Level x Bonus Recipient | - | - | - | - |
|  | Construal Level x Achievement | -2.65 | 1.54 | 4.03 | .045 |
|  | Bonus Recipient x Achievement | -0.37 | 0.80 | 0.22 | .638 |
|  | Construal Level x Bonus Recipient x Achievement | 1.09 | 1.57 | 0.51 | .473 |

|  |  | Regression analysis for randomized response data (RReg) | | | |
| --- | --- | --- | --- | --- | --- |
| Value | Predictor | *b* | *SE b* | *Likelihood Ratio Test* | *p* |
| Power  (10 basic values) | Construal Level | -3.39 | 4.35 | 2.02 | .155 |
|  | Bonus Recipient | 0.06 | 0.76 | 0.01 | .937 |
|  | Power | -0.04 | 0.48 | 0.01 | .932 |
|  | Construal Level x Bonus Recipient | 2.00 | 4.51 | 0.33 | .566 |
|  | Construal Level x Power | 2.73 | 3.04 | 1.86 | .173 |
|  | Bonus Recipient x Power | 0.15 | 0.61 | 0.06 | .813 |
|  | Construal Level x Bonus Recipient x Power | -3.73 | 3.17 | 3.30 | .069 |
| Security  (10 basic values) | Construal Level | -0.55 | 0.85 | 0.42 | .519 |
|  | Bonus Recipient | 0.23 | 0.82 | 0.08 | .782 |
|  | Security | 0.83 | 0.65 | 2.03 | .154 |
|  | Construal Level x Bonus Recipient | -0.80 | 1.30 | 0.39 | .531 |
|  | Construal Level x Security | -1.22 | 0.93 | 1.89 | .169 |
|  | Bonus Recipient x Security | -1.74 | 0.88 | 4.77 | .029 |
|  | Construal Level x Bonus Recipient x Security | 1.87 | 1.44 | 1.31 | .252 |

*Note.* The regression analyses for randomized response data revealed numeric instabilities when estimating the full model for Tradition, Self Direction, and Achievement. We therefore omitted one interaction term (construal level and bonus recipient) that is neither conceptually important nor proved to be empirically relevant in the present study.
