# Supplementary figures and images for: Manipulating the odds: The effects of Machiavellianism and construal level on cheating behavior
Source: PLoS One. 2019 Nov 14;14(11):e0224526. doi: 10.1371/journal.pone.0224526 (PMC6855464; doi:10.1371/journal.pone.0224526)

**Screenshot of the instructions for the coin flip task.**


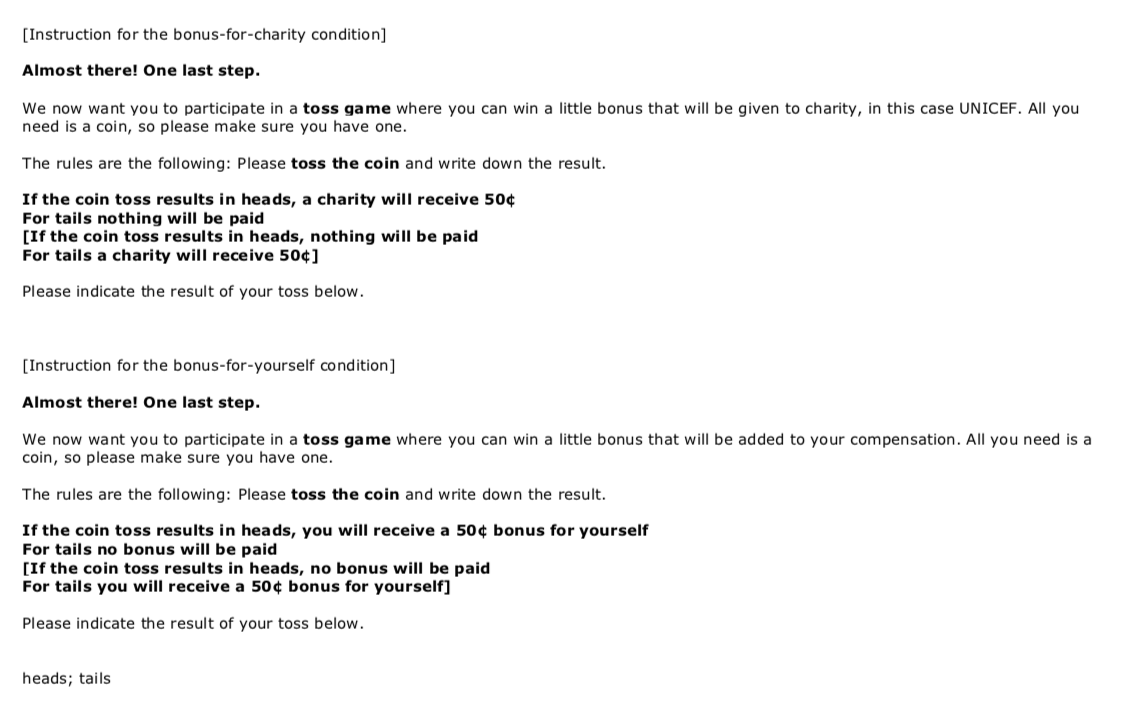

Supplement: S2 Fig — (DOCX) [file pone.0224526.s002.docx]
